# The Nutritional Adequacy and Diet Quality of Vegetarian Menu Substitutions in Urban Kansas Childcare Centers

**DOI:** 10.3390/nu14173464

**Published:** 2022-08-24

**Authors:** Caitlin Jindrich, Jillian Joyce, Elizabeth Daniels, Sandra B. Procter, Kevin Sauer, Jennifer Hanson

**Affiliations:** 1 Mountain Plains Regional Office, Food and Nutrition Service, United States Department of Agriculture, Denver, CO 80204, USA; 2 Department of Food, Nutrition, Dietetics and Health, Kansas State University, Manhattan, KS 66506, USA; 3 Nutritional Sciences, Oklahoma State University, Stillwater, OK 74078, USA

**Keywords:** vegetarian diet, child day care centers, feeding pattern, healthy eating index

## Abstract

While plant-based eating has become increasingly popular, little is known of how this trend has impacted childcare center meals. The purpose of this study was to measure the nutrient content and diet quality of vegetarian alternative lunches and compare these measures to those of standard childcare lunches and nutrient benchmarks representing one-third of the Dietary Reference Intake for 3-year-olds and 4–5-year-olds. Menu data were obtained from seven urban Kansas childcare centers participating in the Child and Adult Care Food Program and regularly providing a vegetarian alternative lunch. The centers provided detailed menu information for 27 days’ worth of meals. The most common vegetarian substitution was cheese, which was used to fulfill all or part of the meat/meat alternative requirement in over three-quarters of the vegetarian alternative meals (*n* = 22). Compared to the standard meals, the vegetarian alternative meals were higher in calories, fat, saturated fat, calcium, and sodium and lower in protein, choline, and diet quality (*p* = 0.05). Both lunch options met the benchmarks for vitamin A, vitamin D, vitamin B12, calcium, and protein. Iron content for both (95% CI: standard 1.61–2.17 mg; vegetarian 1.37–2.7 mg) was below the benchmark. Although additional research is needed to better understand how vegetarianism has impacted childcare meals in the U.S., important differences in the nutrient contents were observed between the standard and vegetarian alternative meals. In addition, the results suggest vegetarian alternative meals that rely heavily on cheese may be of lower diet quality.

## 1. Introduction

While plant-based eating patterns have been observed throughout the course of history [1], the number of individuals that practice vegetarianism currently appears to be increasing [2]. In the United States (U.S.), an estimated 6% of the adult population never consumes meat, fish, seafood, or poultry [3]. Worldwide, the prevalence of vegetarianism varies by country, with rates as high as 30% in India and as low as 1% in Spain [2]. In a representative sample from Germany, 3.4% of the 6–17-year-olds followed a vegetarian diet in 2015–2017, and a comparison to earlier estimates suggests the prevalence is increasing [4].

While the current rate of vegetarianism among U.S. children is unknown, vegetarianism has been described as a social identity rather than simply a way of eating [5]. Given the importance they likely place on their dietary choices, the notion that many vegetarian parents wish to raise their children following a vegetarian eating pattern [6] does not seem unreasonable. A survey of families in Italy revealed that 4.7% of the participating families followed a vegetarian weaning regimen [7]. In the U.S., 5% of respondents to a recent survey identified vegetarian/vegan as a top priority when choosing a family meal [8].

With parents’ interest in vegetarianism appearing to grow, requests for vegetarian options will likely become increasingly common for foodservice operations that serve children. Indeed, middle school foodservice staff perceived vegetarianism as influencing students’ food choices at school [9], and within one U.S. school district, one meatless lunch option, the yogurt and cheese kit, was found to be the most liked lunch option served [10]. While research regarding vegetarian meals within child nutrition programs is scarce, 60% of the childcare centers in our recent survey provided vegetarian alternative meals on a weekly basis [11].

A vegetarian eating pattern can be nutritionally adequate [12,13], as well as healthful [12], and well-planned vegetarian diets have been deemed appropriate for all stages of life [12]. However, in planning vegetarian meals for children, added attention is required in order to assure the adequate intake of nutrients such as iron, zinc, and vitamin B-12 [12]. Unfortunately, vegetarian menu planning may not be receiving the attention it should. Within the U.S., the Child and Adult Care Food Program (CACFP) provides guidance and reimbursement to assist childcare centers in providing nutritious foods that contribute to the wellbeing of children [14]. Although the CACFP meal pattern eligibility requirements are detailed, the flexibility allowed under the rules has the potential to result in dietary imbalances [15]. What is more, a national standard requiring childcare menus undergo a review by a nutrition professional does not exist in the U.S., and relatively few centers rely on a nutrition professional for menu planning [11,16,17,18].

Within the U.S., the rules governing childcare food and nutrition practices differ by state [16,19]. Although participation in CACFP has been linked to improvements in meals and dietary intake, not all centers are eligible or interested in participating. In addition, nutrition-related practices appear to vary between urban and rural childcare centers [18]. The rate of vegetarianism among adults also appears to vary between urban (9%) and rural settings (4%) [3]. Given these differences, as well as the higher rate of vegetarianism among adults in urban areas, the logical setting for this study was a single state’s urban CACFP-participating childcare centers. As such, the purpose of this study was to assess the nutritional adequacy and diet quality of vegetarian alternative lunches within urban Kansas childcare centers participating in CACFP. The nutritional adequacy and diet quality were assessed by comparing the nutrient contact and diet quality scores for vegetarian alternative lunches to those of standard (i.e., meat-containing) childcare lunches, as well as to national nutrition benchmarks.

## 2. Materials and Methods

### 2.1. Design

Data collection occurred during the second phase of a two-phase, cross-sectional study. In phase one, an online survey was used to explore vegetarian menu substitution practices occurring within childcare centers and to also identify centers regularly providing such substitutions for inclusion in phase two. Potential phase one participants were identified using the Kansas State Department of Education (KSDE) public roster of all the state’s childcare centers participating in CACFP [20]. The phase one survey was electronically distributed to the email contact listed for each of the centers located within Kansas’s urban areas. Eight urban areas were identified within Kansas based on the 2010 U.S. Census [21], including Kansas City, Lawrence, Manhattan, Olathe, Overland Park, Shawnee, Topeka, and Wichita. Phase one details and findings have been reported elsewhere [11]. Prior to implementation, Kansas State University’s Committee on Research Involving Human Subjects approved both phases of this study (Proposal #10012 and modification #10012.1).

### 2.2. Data Collection

In order for centers to be included in the second phase of the study, they must have completed the survey, served vegetarian alternative entrees at least three times per week, and agreed to four unannounced phone calls over the course of one month for menu data collection. For the purposes of this study, the definition of vegetarian is that of a lacto-ovo vegetarian dietary pattern. Facilities that participated in menu data collection were given CACFP-compliant recipes and offered a $50 Amazon gift card to purchase foodservice equipment. Unannounced phone calls over the course of 4–6 weeks were used to gather details regarding both the standard meals and the vegetarian meal substitutions served per any given day.

### 2.3. Nutrient Analysis

Once menu data collection was complete, nutrient analysis took place by entering each of the reported meals into Food Processor^®^ Nutrition Analysis software (version 11.2.23, ESHA Research, Salem, OR, USA). Portion sizes were entered into Food Processor based on guidelines for CACFP’s reimbursable lunch meal for 3–5-year-old children [22]. Reported non-creditable items such as sauces and condiments were included in the nutrient analysis. A pre-established codebook [15] was utilized for reported foods that were comparable; otherwise, the closest match was located in the database. When meals served contained mixed-component foods, CACFP-creditable recipes were used. In doing so, some meal components may have portion sizes larger than the minimum requirements for 3–5-year-olds. For example, a creditable cheese pizza recipe includes 1.5 oz of grain in order for the 1.5 ounces of melted cheese to be safely and palatably served. In one instance, the added sugar content was manually reduced by half, as the center reported serving no added sugar baked beans and a matching food product was not available in ESHA’s database. For the purpose of this analysis, low-fat, plain milk fulfilled the fluid milk component for all main and vegetarian meals.

In the instance of incomplete nutrient profiles of a food within Food Processor, missing nutrient values were located using product labels and the USDA’s FoodData Central [23]. See supplemental information in Table A1 (Appendix A) for the methods used to find missing micronutrients unavailable from food labels, Food Processor, or FoodData Central.

### 2.4. Nutrient Benchmarks

As was the practice in prior studies evaluating childcare lunch meals [15,24], one-third of the Dietary Reference Intake (DRI) [25,26] was used as the benchmark for evaluating the lunch meals in this study. The CACFP age group of 3–5-year-olds includes two DRI age categories; therefore, the energy and nutrient benchmark values were determined separately for 3-year-olds and 4 to 5-year-olds. For energy, protein, carbohydrate, and total fiber, the benchmark values were one-third the DRI, with the physical activity levels ranging from sedentary to active [25]. The Acceptable Macronutrient Distribution Range (AMDR) [25] was used as the benchmark value for fat, with the benchmark for saturated fat coming from the Dietary Guidelines for Americans (DGA) [27] recommendation of less than ten percent of the total energy coming from saturated fat.

### 2.5. Diet Quality

The nutrient analysis results were then utilized to score the menus using the Healthy Eating Index-2015 (HEI-2015) [28]. Menus can receive a HEI-2015 maximum score of 100 based on nine categories for adequacy (higher scores for greater offerings) and four categories for moderation (higher scores for less offerings). The higher the score, the more aligned the menu is with the 2015–2020 DGA [27]. Index category scores for total kilocalories, saturated fat, fatty acid ratio, sodium, and added sugars were calculated based on the nutrient values for each menu. The remaining index categories (i.e., total fruits, whole fruits, total vegetables, green and beans, whole grains, dairy, total protein foods, seafood and plant proteins, and refined grains) were scored by totaling the food items served, then comparing the total to the maximum and minimum standard for scoring. The sum of all category scores resulted in the total HEI-2015 score.

### 2.6. Statisical Analyses

Descriptive statistics were used to quantify the nutrient content and HEI-2015 scores of both menu types (i.e., vegetarian alternate and standard meat-containing). Values for several of the outcome variables (i.e., nutrient and HEI score values) were non-normally distributed as assessed by the Shapiro–Wilk test. However, based on the Central Limit Theorem, by which a sample size of roughly 25 or 30 is thought to usually be adequate for a good approximation [29], as well as the relatively robust performance of *t*-tests [30] and the equal sample sizes [30], Student’s *t*-tests (*p* ≤ 0.05) were deemed appropriate to detect differences in the nutrient content and HEI-2015 scores based on meal type. Mean values and 95% confidence intervals (CI) for each nutrient analyzed were compared to the benchmark values for 3-year-olds and 4 to 5-year-olds. For all nutrients except sodium and saturated fat, the menus were deemed adequate if the mean value exceeded the nutritional benchmark for a single value benchmark (e.g., 43 g) or exceeded the lower end for a benchmark with a range (e.g., 373.7–480.0 kcals). For sodium, menus were deemed as meeting the benchmark if the mean value was below the benchmark. For saturated fat, menus were deemed as meeting the benchmark if the mean value was below the upper end of the benchmark range. Statistical analyses were performed using IBM SPSS (version 27.0, IBM Corporation Armonk, NY, USA). 

## 3. Results

Of the 85 childcare centers that completed the phase one survey, seven met the inclusion criteria and provided detailed menu information for a total of 27 days’ worth of standard and vegetarian alternative lunches yielding a total of 54 meals. One additional center’s menus did not meet CACFP guidelines, and therefore, meals from this center were not included in the study. In all, the seven participating centers represented the Kansas urban areas as follows: Lawrence (3), Topeka (2), Olathe (1), Overland Park (1), Kansas City (0), Manhattan (0), Shawnee (0), and Wichita (0).

Vegetarian meal substitutions included cheese, beans, vegetarian nuggets and patties, sunflower seed butter, and tofu. The most common vegetarian substitution was cheese, which was used to fulfill all or part of the meat/–meat alternative requirements in over three-quarters of the vegetarian alternative meals (*n* = 22). The most common meat components served was poultry (*n* = 13), which was used to fulfill all or part of the meat/meat alternative requirements in nearly one-half of the meals (*n* = 13). See Table 1.

### 3.1. Nutritional Adequacy

The mean nutrient values for the standard and vegetarian alternative lunch menus are given in Table 2. The vegetarian alternative menus were higher in calories, fat, saturated fat, calcium, and sodium. The standard menus were higher in protein and choline (Table 2).

The nutrients that met the benchmark for both age groups in both menu types included protein, vitamin A, calcium, vitamin B12, and zinc (Table 3 and Table 4). The nutrients that did not meet the benchmarks for both age groups and menus included iron, fiber, potassium, and sodium (Table 3 and Table 4). Choline was below the benchmark for the vegetarian alternative menu, and folate was below the benchmark for 4 to 5-year-olds in the standard menu. Both the standard and the vegetarian alternative menus provided less than one-third of the day’s calories and carbohydrates, and the vegetarian alternative menus did not meet the benchmark for saturated fat.

### 3.2. Diet Quality

The vegetarian alternative meal HEI-2015 scores ranged 48.9–89.4, with a mean score of 64.9. The standard meal HEI-2015 scores ranged 58.0–92.0, with a mean score of 71.8. Compared to the standard meals, the HEI-2015 scores were lower for the alternative vegetarian meals, t (44.7) = −2.14, *p* = 0.038 (Table 5). Seventeen vegetarian alternative meals received a score of zero for the total protein component when cheese was served as the meat alternative. Both menu types received the maximum points for dairy for all meals. All but one meal received the maximum score for added sugar, and all but two meals received the maximum score for refined grains (Table 6). Across all the menus evaluated, the HEI-2015 component scores were consistently low for greens and beans, seafood and plant proteins, and fatty acid ratio components.

## 4. Discussion

This study aimed to assess the nutrient adequacy and diet quality of vegetarian alternative lunches within urban Kansas childcare centers. The results identified many important differences between the standard and vegetarian alternative meals served in urban Kansas childcare centers. However, it is important to view these differences in the context of the national data and benchmarks for each nutrient.

Both menus failed to meet one-third of the DRI for iron in both age categories. Notably, the difference between the mean iron content in vegetarian alternative and standard meat-containing menus was nonsignificant. This lack of a significant difference is noteworthy, because the iron intake is often cited as a vegetarian nutrient of concern [12,31]. Further exploration of the iron content between omnivore and vegetarian meal patterns within federal child nutrition programs is warranted. The protein, another vegetarian nutrient of concern, was higher in the standard meals. However, both menu types provided protein well beyond the DRI.

Diets in the U.S. are low in potassium and fiber [31]. This analysis of childcare menus found that both the standard meat-containing and vegetarian alternative menus provided less than one-third of the DRI for both nutrients. Overall, U.S. diets are also low in choline across all ages, and one-third of 2 to 3-year-olds and over one-half of 4 to 5-year-olds do not meet the adequate intake for this nutrient [32]. In this study, the standard meals were higher in choline compared to the vegetarian alternative meals. The standard meals met the choline benchmark for both 3-year-olds and 4 to 5-year-olds. However, the vegetarian alternative menus did not meet the choline benchmark. Including more choline-rich foods such as eggs could help improve the choline content of the vegetarian meals.

When compared to the national average of HEI-2015 component scores for 2–18- year-olds, the average for each of the menu types in this study had better scores for the total fruits, total vegetables, green and beans, whole grains, dairy, seafood and plant proteins, refined grains, sodium, and added sugars. Whole fruit scores were equal to the national average, and fatty acid scores were worse. Saturated fat scores were higher for the standard meals and lower for the vegetarian meals, meaning the vegetarian alternative meals contained higher levels of saturated fat than the meat-containing meals. Both the standard and vegetarian alternative meals had higher total HEI-2015 scores compared to the national average from NHANES data for 2–5-year-olds [33].

The vegetarian alternative menus had a wide range of HEI-2015 scores. Unlike the CACFP [14], neither the DGA nor the HEI-2015 recognize cheese as a protein food [28,31]. Instead, both the DGA and the HEI-2015 categorized cheese as a dairy food. Therefore, the vegetarian menus received zero HEI points for total protein on days in which cheese fulfilled the meat/meat alternate component. Both the standard and vegetarian alternative meals consistently received the maximum dairy component scores based on the fluid milk component alone. Thus, cheese did not improve the vegetarian alternative meal dairy component scores. Overall, the vegetarian alternative meals routinely received lower scores for saturated fat and the sodium content compared to the standard meals. On days when cheese was served as the meat alternative, the total HEI-2015 score for the meal was relatively low. When vegetarian entrees such as beans or nut butter were served as the meat alternative component, the total HEI-2015 score for the meal was much higher. Though five of the six meat alternative options allowable were represented in the 27 days of vegetarian menus collected, cheese was used to fulfill all, or a portion of, the meat alternative in 81.5% (*n* = 22) of the qualifying CACFP vegetarian meals. Future research should explore barriers to serving a larger variety of meat alternative options in the child-care setting.

Like the Academy of Nutrition and Dietetics’ stance on childcare centers limiting added sugars and refined grains [34], the childcare menus evaluated in this study met this recommendation. Only one of the 54 meals evaluated did not receive the maximum score for limiting added sugars; notably, barbecue sauce was served as a condiment on this day. Although non-creditable foods, such as condiments, can be served with meals, CACFP’s Optional Best Practices [35] recommends limiting non-creditable foods that are sources of added sugars. The menus evaluated in this analysis meet this Optional Best Practices recommendation.

The sodium and fatty acid ratio categories of both menus received low HEI-2015 scores, indicating these categories did not align well with the DGA [27] or with the Academy of Nutrition and Dietetics’ recommendation for benchmarks for nutrition in childcare [21]. The means for both menu types exceeded one-third of the DRI for sodium for both age ranges, which is consistent with previous research that identified higher than recommended sodium levels in Oklahoma childcare settings [36]. Despite attempts to lower the sodium targets in child nutrition programs, the implementation of such targets has been delayed [37] as programs reported difficulty finding products that comply [38]. It is important to note that, at this time, CACFP does not provide stipulations on the sodium content for reimbursable meals, though such stipulations would be beneficial and warrant consideration. In addition to sodium, the Academy of Nutrition and Dietetics recommends limiting saturated fatty acids in childcare nutrition. The results show that the standard meals had lower levels of saturated fatty acids than the vegetarian alternative meals. Nearly two-thirds of the vegetarian alternative menus served cheese as the meat alternative component. A creditable meat alternate serving of cheese for a reimbursable meal is one-and-a-half ounces. One-and-a-half ounces of reduced fat cheddar provides approximately 119.2 kcal, 4.92 g saturated fat, and 308.3 mg sodium, likely explaining why the vegetarian alternative menus provided significantly more of these nutrients. Future evaluations of nutrient and diet quality analyses comparing menus utilizing cheese versus menus utilizing other allowable meat alternative components are warranted.

Over the last 40 years, cheese consumption in the U.S. has more than doubled [39]. Although the underlying reasons for the extensive use of cheese as a meat alternative are unknown, consumer preferences, such as the apparent popularity of cheese [10,39], likely play a role. Other factors potentially contributing to the heavy use of cheese include: (a) the prevalence of peanut allergies [40], which has prompted many centers to stop using peanut butter as a meat alternative, (b) food storage space limitations [18], which may deter childcare centers from purchasing meat alternative items such as soy products and pulses that they would otherwise not have on hand, (c) menu planning completed without the oversight of a nutrition professional [11,16,17,18], and (d) the potential confusion created by the unclear manner in which pulses have been grouped with various food groups (e.g., vegetables and meat alternatives) in the DGA [41]. While our previously reported findings indicate a lack of confidence surrounding CACFP and vegetarian meal alternatives [11], additional research is needed to confirm the underlying reasons for the extensive use of cheese as a vegetarian meat alternative.

The limitations of this study are important to note. Although all qualifying centers were included in the menu analysis, not all urban areas were represented in this phase of the study. Furthermore, the exclusion of childcare centers from the rural areas of Kansas resulted in a sample that was not representative of all the centers across the state. Similarly, the results may not be generalizable to areas outside of Kansas.

The limitations of the menu analysis include unknown brand/manufacturers of products, recipes, preparation, and cooking methods, akin to Frampton et al. [36]. Additionally, only one meal of the day was analyzed; thus, nutrients the menus provided in excess or inadequate amounts cannot be generalized to the entire day’s or week’s worth of intake. Database limitations were present within the ESHA Food Processor, with previously described methods used to compensate for such limitations [15].

The optimal growth and development during childhood is reliant on a healthful eating pattern. Although consumption of the lunch meals was not measured, the nutritional quality of what children eat while in childcare has been shown to be associated with that of the foods they are served [42]. Childhood eating habits that are healthy and that reflect the DGA set the foundation for lifelong patterns that reduce the risk of diet-related chronic disease [31]. As such, careful menu planning plays an import role in the health and wellbeing of children attending childcare. While this study is the first of its kind, subsequent research is needed to better assess the nutrient content and diet quality of vegetarian childcare meals from across all regions of the U.S.

## 5. Conclusions

In conclusion, the analyzed urban Kansas childcare center CACFP menus for 3–5 year-olds have higher diet quality scores than the national average for the diets of U.S. children. Notably, there is limited use of the allowable meat alternative components beyond cheese. Additionally, both menu types analyzed could be improved upon by the inclusion of more iron-dense foods. The vegetarian meals could be improved upon by using less cheese and using more plant-based alternatives such as lentils, beans, and soy, which are good sources of protein but are also low in saturated fat. The further evaluation of limiting sodium and of limiting the use of cheese as a meat alternative within CACFP should be performed. Such changes would better align CACFP childcare menus with the DGA recommendations.

## Figures and Tables

**Table 1:** Frequency of meat/meat alternates served across 54 total lunches.

Meat/Meat Alternates	Frequency
**Standard**	***n* = 27**
Poultry (chicken and turkey)	10
Beef	7
Fish	2
Turkey and cheese	3
Tuna and cheese	2
Cheese and pepperoni	1
Beef and beans	1
Hot dogs and beans	1
**Vegetarian**	***n* = 27**
Cheese	17
Cheese & beans	3
Cheese and vegetarian meat alternative product	2
Vegetarian meat alternative product	2
Sunflower seed butter	1
Beans	1
Tofu	1

**Table 2:** A *t*-test comparison of the standard and vegetarian alternative lunch menu nutrient values.

	Mean Nutrient Values (Range)	*t*-test Statistic	*p*	
Nutrient	Standard (*n* = 27)	Vegetarian Alternative (*n* = 27)		
Energy (kcal)	295.6 (260.4–330.8)	328.8 (271.1–386.5)	−2.56	0.014 *
Protein (g)	20.5 (17.6–23.3)	18.8 (16.2–21.4)	2.22	0.031 *
Carbohydrate (g)	34.8 (28.0–41.6)	36.8 (28.6–45.1)	−0.99	0.329
Fat (g)	8.9 (6.4–11.3)	12.7 (6.9–18.4)	−3.15	0.003 ^a^ *
Saturated fat (g)	3.5 (2.5–4.5)	5.9 (3.2–8.5)	−4.36	<0.001 ^a^ *
Monounsaturated fat (g)	2.8 (1.6–4.1)	3.9 (0.6–7.3)	−1.61	0.115
Polyunsaturated fat (g)	1.4 (0.3–2.5)	1.7 (0.6–2.9)	−0.91	0.366
Fiber (g)	3.6 (2.3–4.9)	4.4 (2.2–6.5)	−1.47	0.148
Folate DFE (µg)	57.3 (32.7–81.8)	70.2 (39.2–101.2)	−1.70	0.095
Vitamin A RAE (µg)	218.2 (117.6–318.9)	272.8 (172.3–373.2)	−1.99	0.052
Calcium (mg)	333.1 (248.3–417.9)	555.7 (360.9–750.4)	−5.44	<0.001 ^a^ *
Vitamin B12 (µg)	1.4 (1.0–1.8)	1.4 (1.0–1.7)	−0.06	0.952
Zinc (mg)	2.6 (1.7–3.6)	2.7 (2.2–3.1)	−0.12	0.905 ^a^ *
Potassium (mg)	671.8 (582.7–760.9)	634.4 (504.5–764.2)	1.23	0.223
Iron (mg)	1.8 (1.2–2.4)	1.7 (1.0–2.5)	0.42	0.676
Sodium (mg)	523.5 (323.9–723.1)	692.9 (470.6–915.3)	−2.95	0.005 *
Choline (mg)	82.88 (69.94–95.82)	65.9 (56.16–75.64)	5.45	<0.001 *

^a^ Equal variance not assumed. * Statistical significance at *p* < 0.05.

**Table 3:** Nutritional adequacy of the standard menus.

Standard Menus					
Nutrient	Nutrient	Nutrient	Nutrient	Nutrient	Nutrient
Energy (kcal)	295.6	35.2	281.6–309.5	373.7–480.0 *	401.0–521.3 *
Protein (g)	20.5	2.9	19.3–21.6	4.9	5.4
Carbohydrate (g)	34.8	6.9	32.1–37.5	43 *	43 *
Fat (g)	8.9	2.5	7.9–9.8	12.5–21.3 *	11.1–20.3 *
Saturated Fat (g)	3.5	1.00	3.1–3.9	4.2–5.3	4.5–5.8
Fiber (g)	3.6	1.3	3.1–4.2	5.2–6.7 *	5.6–7.3 *
Folate DFE (µg)	57.3	24.5	47.5–67.0	50	66 *
Vitamin A RAE (µg)	218.2	100.6	178.4–258.1	100	132
Calcium (mg)	333.1	84.8	299.6–366.7	165	264
Vitamin B12 (µg)	1.4	0.4	1.2–1.5	0.3	0.4
Zinc (mg)	2.6	0.9	2.3–3.0	1.0	1.7
Potassium (mg)	671.8	89.1	636.5–707.0	1000 *	1254 *
Iron (mg)	1.8	0.6	1.6–2.0	2.3 *	3.3 *
Sodium (mg)	523.5	199.6	444.5–602.4	333 *	396 *
Choline (mg)	82.9	12.9	77.8–88.0	66	82.5

* Mean value did not meet the benchmark. For energy, protein, carbohydrate, and total fiber, the benchmark is one-third the DRI, with physical activity levels ranging from sedentary to active. The Acceptable Macronutrient Distribution Range (AMDR) was used as the benchmark for fat. The saturated fat benchmark of <10% was based on the DGA.

**Table 4:** Nutritional adequacy of the vegetarian menus.

Vegetarian Menus					
Nutrient	Mean	SD	95% CI	3-Year-Olds’ Benchmark	4 to 5-Year-Olds’ Benchmark
Energy (kcal)	328.8	57.7	306.0–351.6	373.7–480.0 *	401.0–521.3 *
Protein (g)	18.8	2.6	17.8–19.8	4.9	5.4
Carbohydrate (g)	36.8	8.2	33.6–40.1	43 *	43 *
Fat (g)	12.7	5.8	10.4–14.9	12.5–21.3	11.1–20.3
Saturated Fat (g)	5.9	2.6	4.8–6.9	4.2–5.3 *	4.5–5.8 *
Fiber (g)	4.4	2.2	3.5–5.2	5.2–6.7 *	5.6–7.3 *
Folate DFE (µg)	70.2	31.0	57.9–82.5	50	66
Vitamin A RAE (µg)	272.8	100.5	233.0–312.5	100	132
Calcium (mg)	555.7	194.7	478.6–632.7	165	264
Vitamin B12 (µg)	1.4	0.3	1.2–1.5	0.3	0.4
Zinc (mg)	2.7	0.5	2.5–2.8	1.0	1.7
Potassium (mg)	634.4	129.9	583.0–685.7	1000 *	1254 *
Iron (mg)	1.7	0.8	1.4–2.0	2.3 *	3.3 *
Sodium (mg)	692.9	222.3	605.0–780.9	333 *	396 *
Choline (mg)	65.9	9.7	62.1–69.8	66 *	82.5 *

* Mean value did not meet the benchmark. For energy, protein, carbohydrate, and total fiber, the benchmark is one-third the DRI, with physical activity levels ranging from sedentary to active. The Acceptable Macronutrient Distribution Range (AMDR) was used as the benchmark for fat. The saturated fat benchmark of <10% was based on the DGA.

**Table 5:** A *t*-test comparison of the standard and vegetarian alternative HEI-2015 scores.

		Mean Score (Range)		*t*-test Statistic	*p*
HEI-2015 Category	Max Score	Standard (*n* = 27)	Vegetarian Alternative (*n* = 27)		
Total Fruit	5	4.35 (0.00–5.00)	4.15 (0.00–5.00)	0.46	0.650
Whole Fruit	5	4.44 (0.00–5.00)	4. 44 (0.00–5.00)	0.00	1.000
Total Vegetables	5	4.46 (3.24–5.00)	4.34 (2.79–5.00)	0.61	0.543
Dark Greens and Legumes	5	2.04 (0.00–5.00)	2.59 (0.00–5.00)	−0.81	0.423
Whole Grains	10	8.63 (0.00–10.00)	8.43 (0.00–10.00)	0.21	0.843
Dairy	10	10.0 (10.0–10.0)	10.0 (10.0–10.0)	-- ^a^	-- ^a^
Total Protein Foods	5	4.91 (2.69–5.00)	1.85 (0.00–5.00)	6.37	<0.001 ^b^ *
Seafood & Plant Proteins	5	1.48 (0.00–5.00)	1.85 (0.00–5.00)	−0.57	0.572
Fatty Acids	10	1.69 (0.00–7.00)	1.67 (0.00–10.00)	0.02	0.987
Refined Grains	10	9.93 (9.00–10.00)	9.96 (9.00–10.0)	−0.59	0.561
Sodium	10	3.89 (0.00–10.00)	2.33 (0.00–10.00)	1.63	0.108
Added Sugars	10	9.89 (7.00–10.00)	10.0 (10.0–10.0)	−1.00 ^b^	0.322 ^b^ *
Saturated Fats	10	6.06 (1.2010.00)	3.24 (0.00–10.00)	3.06	0.004 ^b^ *
HEI−2015 Total	100	71.77 (57.50–91.99)	64.87 (48.00–89.40)	2.14 ^b^	0.038 ^b^ *

^a^*t* value could not be computed, because the standard deviations of both groups are 0, ^b^ equal variance not assumed, and * statistical significance at *p* < 0.05.

**Table 6:** Daily maximum HEI-2015 component scores among the standard meal and vegetarian alternative HEI-2015 component scores.

HEI-2015 Category	Maximum Score Possible	Proportion of Days Achieving Maximum Score Possible		
		Standard (*n* = 27)	Vegetarian Alternative (*n* = 27)	All Menus Combined (*n* = 54)
Total Fruits	5	63.0% (*n* = 17)	37.0% (*n* = 10)	50.0% (*n* = 27)
Whole Fruits	5	88.9% (*n* = 24)	88.9% (*n* = 24)	88.9% (*n* = 48)
Total Vegetables	5	48.1% (*n* = 13)	59.3% (*n* = 16)	53.7% (*n* = 29)
Dark Greens and Legumes	5	40.7% (*n* = 11)	51.9% (*n* = 14)	46.3% (*n* = 25)
Whole Grains	10	85.2% (*n* = 23)	77.8% (*n* = 21)	81.5% (*n* = 44)
Dairy	10	100% (*n* = 27)	100% (*n* = 27)	100% (*n* = 54)
Total Protein Foods	5	96.3% (*n* = 26)	37.0% (*n* = 10)	66.7% (*n* = 35)
Seafood and Plant Proteins	5	29.6% (*n* = 8)	37.0% (*n* = 10)	33.3% (*n* = 18)
Fatty Acids	10	0% (*n* = 0)	7.4% (*n* = 2)	3.7% (*n* = 2)
Refined Grains	10	92.6% (*n* = 25)	96.3% (*n* = 26)	94.4% (*n* = 51)
Sodium	10	14.8% (*n* = 4)	3.7% (*n* = 1)	9.3% (*n* = 5)
Added Sugars	10	96.3% (*n* = 26)	100% (*n* = 27)	98.1% (*n* = 53)
Saturated Fats	10	14.8% (*n* = 4)	14.8% (*n* = 4)	14.8% (*n* = 8)

## Data Availability

The data presented in this study are available on request from the corresponding author.

## Appendix A

**Table A1:** Micronutrient substitution methods.

Food	Nutrient	Substitution
Breadcrumbs, panko, plain	Vitamin B12, folate, choline, zinc	Wheat flour, white, all-purpose, enriched, bleached, energy match
Breadcrumbs, panko, whole wheat	Vitamin B12, folate, choline, zinc, potassium	Wheat flour, whole grain, energy match
Potato wedges	Choline	Potato, baked
Potato wedges	Folate	Potato, steak fries
Ranch salad dressing, reduced fat	Choline	Ranch dressing (regular), energy matched
Sunflower seed butter	Choline	Sunflower seeds, energy matched
Tortilla chips, nacho, with enriched masa	Choline	Corn flour, masa, enriched, white, energy match
Whole wheat breading on nuggets	Energy, protein, carbohydrate, fat, calcium, vitamin B12, folate, vitamin A, choline, zinc, potassium, iron, sodium	Crackers, saltine, whole wheat
